# The Drivers of Acceptance of Artificial Intelligence–Powered Care Pathways Among Medical Professionals: Web-Based Survey Study

**DOI:** 10.2196/33368

**Published:** 2022-06-21

**Authors:** Lisa Cornelissen, Claudia Egher, Vincent van Beek, Latoya Williamson, Daniel Hommes

**Affiliations:** 1 Faculty of Science Athena Institute Vrije Universiteit Amsterdam Amsterdam Netherlands; 2 Faculty of Health Medicine and Life Sciences Maastricht University Maastricht Netherlands; 3 DEARhealth Amsterdam Netherlands; 4 Department of Gastroenterology & Hepatology Leiden University Medical Center Leiden Netherlands

**Keywords:** technology acceptance, artificial intelligence, health care providers, machine learning, technology adoption, health innovation, user adoption

## Abstract

**Background:**

The emergence of Artificial Intelligence (AI) has been proven beneficial in several health care areas. Nevertheless, the uptake of AI in health care delivery remains poor. Despite the fact that the acceptance of AI-based technologies among medical professionals is a key barrier to their implementation, knowledge about what informs such attitudes is scarce.

**Objective:**

The aim of this study was to identify and examine factors that influence the acceptability of AI-based technologies among medical professionals.

**Methods:**

A survey was developed based on the Unified Theory of Acceptance and Use of Technology model, which was extended by adding the predictor variables perceived trust, anxiety and innovativeness, and the moderator profession. The web-based survey was completed by 67 medical professionals in the Netherlands. The data were analyzed by performing a multiple linear regression analysis followed by a moderating analysis using the Hayes PROCESS macro (SPSS; version 26.0, IBM Corp).

**Results:**

Multiple linear regression showed that the model explained 75.4% of the variance in the acceptance of AI-powered care pathways (adjusted *R*^2^=0.754; *F*_9,0_=22.548; *P*<.001). The variables medical performance expectancy (*β*=.465; *P*<.001), effort expectancy (*β*=–.215; *P*=.005), perceived trust (*β*=.221; *P*=.007), nonmedical performance expectancy (*β*=.172; *P*=.08), facilitating conditions (*β*=–.160; *P*=.005), and professional identity (*β*=.156; *P*=.06) were identified as significant predictors of acceptance. Social influence of patients (*β*=.042; *P*=.63), anxiety (*β*=.021; *P*=.84), and innovativeness (*β*=.078; *P*=.30) were not identified as significant predictors. A moderating effect by gender was found between the relationship of facilitating conditions and acceptance (*β*=–.406; *P*=.09).

**Conclusions:**

Medical performance expectancy was the most significant predictor of AI-powered care pathway acceptance among medical professionals. Nonmedical performance expectancy, effort expectancy, perceived trust, and professional identity were also found to significantly influence the acceptance of AI-powered care pathways. These factors should be addressed for successful implementation of AI-powered care pathways in health care delivery. The study was limited to medical professionals in the Netherlands, where uptake of AI technologies is still in an early stage. Follow-up multinational studies should further explore the predictors of acceptance of AI-powered care pathways over time, in different geographies, and with bigger samples.

## Introduction

Health care systems are currently burdened owing to an aging population, increasing life expectancy, the development of expensive therapies, an inefficient design, and a growing demand for a good quality of care [1]. This has resulted in rising health care expenditure and threatened the accessibility of care [1]. Artificial Intelligence (AI), broadly defined as the capability of a machine to imitate intelligent human behavior [2], has the potential to help improve many of these challenges. Through the development of sophisticated algorithms, AI can assist in the diagnosis, monitoring, and treatment of patients, it can help streamline services and render administrative tasks more efficient [3]. Even though AI has already been proven beneficial in several health areas, such as clinical decision support, patient monitoring, health interventions, and health care administration [4-6], its impact on health care delivery has thus far remained limited [7].

Several barriers for entry have been identified, which explain the underuse of AI in health care delivery, including regulatory constraints, ethical considerations, lack of transparency, and the lack of facilitating conditions [8-10]. In addition, a crucial barrier for the implementation of AI-based technologies is the lack of adoption among medical professionals [9]. Moreover, individuals’ acceptance and utilization of technologies are proposed to be the most important factors for health technology adoption [11]. Currently, it is poorly understood what the reasons are for medical professionals (not) adopting AI technologies. Recently, some studies have been conducted to research the perspectives of the end users in the implementation of AI-based technologies, but more insight is essential [12].

The lack of understanding as to what informs the resistance among medical professionals in regard to the adoption of AI-based technologies can have important negative consequences, as it can limit and delay substantial improvements in health care delivery and result in wasted research and high design costs. Therefore, this study investigated medical professionals’ perspectives on the adoption of AI-powered care pathways by identifying which factors influence, and to what degree, the acceptability of AI-based technologies among these stakeholders.

This study focused on AI-powered care pathway technology. This technology enables the management of chronic diseases on a digital platform. All stakeholders (including medical professionals, patients, and caregivers), involved medical activities, and associated support programs are included in this platform. It enables medical professionals to constantly monitor their patient population’s disease activity and mental well-being through a patient app. The care pathways were designed to offer the right care at the right time and are continuously risk-adjusted using AI. This risk adjustment is created through several steps. The first step entails updating the patient’s data into the system. In the second step, the data are classified in the patient’s risk profile. In the third step, the risk profile–learning models and algorithms (based on AI) update the care pathway upon the most recent profile of the patient. From the update, a new recommendation is formulated (or not, if no alteration is necessary). Lastly, the medical professional can accept or reject the received recommendation based on various considerations and in dialogue with the patient.

## Methods

### Recruitment

The target population consisted of medical professionals who were employed at a Dutch hospital or other hospital staff who worked on improvement of the quality of care. Participants were mainly invited to participate through the physicians’ network (email, LinkedIn, virtual, and in-person meetings). A web-based survey was created using Qualtrics [13]. The data were gathered between the April 20 and June 1, 2021. The survey took approximately 7 minutes to complete.

### Model

**Figure 1:**
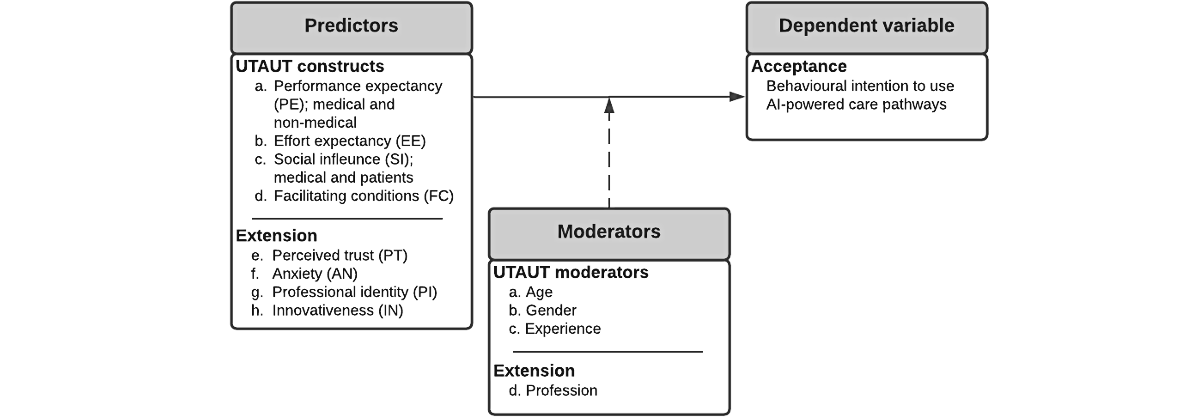
Overview of the conceptual model used in this study. The predictor variables (performance expectancy, effort expectancy, social influence, facilitating conditions, perceived trust, anxiety, professional identity, and innovativeness) are hypothesized to influence the variance the acceptance of AI-powered care pathways. The moderators (age, gender, experience, and profession) are hypothesized to influence the relationship between the predicator variables and the dependent variable. AI: artificial intelligence, UTAUT: Unified Theory of Acceptance and Use of Technology.

To determine factors that influence the acceptability of AI-powered care pathways among medical professionals, the validated Unified Theory of Acceptance and Use of Technology (UTAUT) model was extended and subsequently applied [14]. According to the UTAUT model, the acceptance of new technology can be measured by the behavioral intention (BI) to use a certain technology. The following predictor variables from UTAUT were included for the analysis: performance expectancy (PE; divided into medical and nonmedical), effort expectancy (EE), social influence (SI; divided in to social influence patients and medical), and facilitating conditions (FC) [14]. The original construct performance expectancy was divided into medical and nonmedical since Shaw et al [15] stated that AI-based technologies have different relevant tasks (clinical, epidemiological, and operational), and uncovering the value proposition between these tasks is an essential consideration for successful adoption. To uncover the value proposition for AI-powered care pathways, the construct of performance expectancy medical (*clinical* in article of Shaw et al [15]) and performance expectancy nonmedical (*operational* in article of Shaw et al [15]). The construct of social influence was divided since different studies highlighted that social influence is often studied from one influential group while neglecting influence of other groups [3,4,16,17]. Eckhardt et al [16] proposed to derive relevant influential groups and treat their different impacts with due respect. In light of this study, two main influential groups were identified, namely medical *professionals* and *patients*, resulting in the following constructs: social influence medical experts (SIME) and social influence patients (SIPA). The model was enriched with several variables that relevant scientific literature from multiple disciplines identified as playing a role in shaping the technology acceptance of AI-based technologies. These variables were the following: perceived trust (PT) [18], anxiety (AN) [19], professional Identity (PI) [20], and innovativeness (IN) [21] (Table 1). Furthermore, three moderators from the UTAUT model were included, namely age, gender, and experience [14]. In the original model, experience is defined as experience with the used technology. In the light of this study, this moderator was not applicable since AI-powered care pathways are in a premature stage of implementation. Therefore, the definition was changed to “Years of experience in the medical field“ [22]. An additional moderator, *profession*, was added since different professions have different responsibilities and tasks, which is hypothesized to influence the relationships between the predictor variables and the acceptance. A schematic overview of the model is shown in Figure 1.

**Table 1:** Definitions of the predictor variables for the behavioral intention to use artificial intelligence (AI)-powered care pathways.

Construct	Operational definition
Medical performance expectancy^a^	Degree to which an individual believes that using AI-powered care pathways will help him or her to attain gains in terms of the provided quality of care [14,15]
Nonmedical performance expectancy^a^	Degree to which an individual believes that using AI-powered care pathways will help him or her to attain gains in productivity, efficiency, and communication [14,15]
Effort expectancy	Degree of ease associated with the use of the system [14]
Social influence patients^b^	Degree to which an individual perceives that patients believe that he or she should use the new system [14,16,17]
Social influence medical^b^	Degree to which an individual perceives that other medical organizations or colleagues believe that he or she should use the new system [14,16,17]
Facilitating conditions	Degree to which an individual believes that an organizational and technical infrastructure exists to support the use of the system [14]
Perceived trust	Users’ specific trust that AI-powered care pathways have the ability, integrity, and benevolence in providing their service [18]
Anxiety	The fear (eg, sadness, perception, and stress caused by stress-creating situations) experienced by an individual during their interaction with AI-powered care pathways [19]
Professional identity	The attitudes, values, knowledge, beliefs, and skills that are shared with others within a professional role being undertaken by the individual [23]
Innovativeness	Degree to which an individual is relatively earlier in adopting an innovation than other members of his (social) system [21]

^a^The original determinant in the Unified Theory of Acceptance and Use of Technology (UTAUT) model of performance expectancy was divided in two separate variables since performance expectancy for AI-powered care pathways can be viewed from a medical and nonmedical perspective.

^b^The original determinant in the UTAUT model of social influence was divided in two separate variables since it is hypothesized that patients and medical organizations or colleagues have different influences.

### Survey

The survey contained questions about demographics including age, gender, experience, and profession. Then, the survey participants were invited to rate statements concerning the constructs that needed to be ranked using a 5-point Likert scale (1=totally disagree, 5=totally agree). The survey items were formulated by adopting statements from prior research and by developing new statements within the research group (Multimedia Appendix 1 for statements). Before collecting the data, the survey was tested extensively through a pilot study with graduate students, individuals who were not familiar with AI-powered care pathways, and DEARhealth. staff. This pilot tested for confusing formulations, lay-out problems, the approximate time to complete the survey, and the technical resources needed. Where needed, adjustments were subsequently made.

### Statistical Analysis

#### Measurement Model Testing

To assess the reliability of the measurements, the internal consistency was tested using Cronbach *α* values. The commonly used rule of thumb for Cronbach *α* was used where a value is acceptable above .6, questionable between .5 and .6, and unacceptable below .5 [24]. Methods to try and ensure reliability such as item removal were performed when problems of reliability arose. Furthermore, Pearson correlations between predictor variables were tested to rule out any internal relations, a rule of thumb of >0.7 was used.

#### Relationship Testing

The data were analyzed using SPSS (version 26; IBM Corp) including the extension PROCESS macro developed by [25]. To exclude responses with missing data, a data clean was conducted. A descriptive analysis to get an insight into the respondents' demographic characteristics was conducted. Then, a multiple linear regression analysis was performed to test the contribution of each predictor variable on the variance of BI. Before conducting the multiple linear regression analysis, the assumptions were checked to rule out violations. The checked violations were linearity, multivariate outliers, heteroscedasticity, and multicollinearity. A *P* value of <.01 was considered significant (Multimedia Appendix 2). Lastly, moderation analysis was conducted using PROCESS model 1. The moderation analysis used hierarchical multiple regression with an interaction term.

### Ethical Considerations

No additional ethical approval was needed according to the online check performed using the web-based BETCHIE test of the Beta faculty of Vrije Universiteit Amsterdam, which indicated that the target group was not considered a vulnerable group in this research. The privacy of the respondents was ensured by anonymizing the survey in Qualtrics. The researchers could not track the source of the survey, and no private information was collected. Participating in this research was voluntary.

## Results

### Participants

In total, 111 health care professionals started the survey. After excluding respondents with missing answers (n=41) or monotone answers (n=3), 67 remained. Of the 67 participants, 41 (61.2%) identified as female and 26 (38.8%) as male. The age distribution in this research was the following: <35 years (20/67, 29.9%), 35-55 years (n=27, 40.3%), and >55 years (n=20, 29.9%). For the different medical professions, an overrepresentation of physicians (n=28, 41.8%) was identified as compared to the number of nurses (n=14, 20.9%) and nurse specialists (n=2, 3.0%) (Table 2).

**Table 2:** Participant demographics (N=67).

Characteristics			Participants, n		Participants, %
**Gender**					
	Male	26		38.8	
	Female	41		61.2	
**Age (years)**					
	18-24	5		7.5	
	25-34	15		22.4	
	35-44	16		23.9	
	45-54	11		16.4	
	55-64	17		25.4	
	≥65	3		4.5	
**Experience in the medical field**					
	≤2	5		7.5	
	3-5	10		14.9	
	6-10	7		10.4	
	11-20	19		28.4	
	21-30	14		20.9	
	≥31	12		17.9	
**Profession**					
	Physician	28		41.8	
	Nurse specialist	2		3.0	
	Nurse	14		20.9	
	Management	11		16.4	
	Consultant	11		16.4	
	Other function in hospital	13		19.4	

### Outcomes

#### Measurement Testing Findings

A Cronbach *α* score was calculated for each construct of the model to validate the internal consistency of the measurement statements within the variable (Table 3). The variable of SIME showed a Cronbach *α* below .50 and was removed from the analysis. The Pearson correlation coefficients between any of the predictor variables did not exceed 0.7, indicating an acceptable correlation between the predictors (Multimedia Appendix 3).

**Table 3:** Internal reliability of the constructs based on the 3 statements using Cronbach *α* values. Social influence medical experts (SIME) and facilitating conditions (FCs) showed unacceptable internal consistency (Cronbach *α*<.5). Item removal resulted in a better Cronbach *α* for facilitating conditions.

Variable	Cronbach *α*
Innovativeness	.706
Anxiety	.701
FC→FC1+FC2 (item removal of FC3)	.455 → .512
Nonmedical performance expectancy	.662
Social influence patients	.667
Medical performance expectancy	.638
Social influence medical experts	.244
Professional identity	.748
Perceived trust	.717
Effect expectancy	.816
Behavioral intention	.916

#### Regression Outcomes

**Figure 2:**
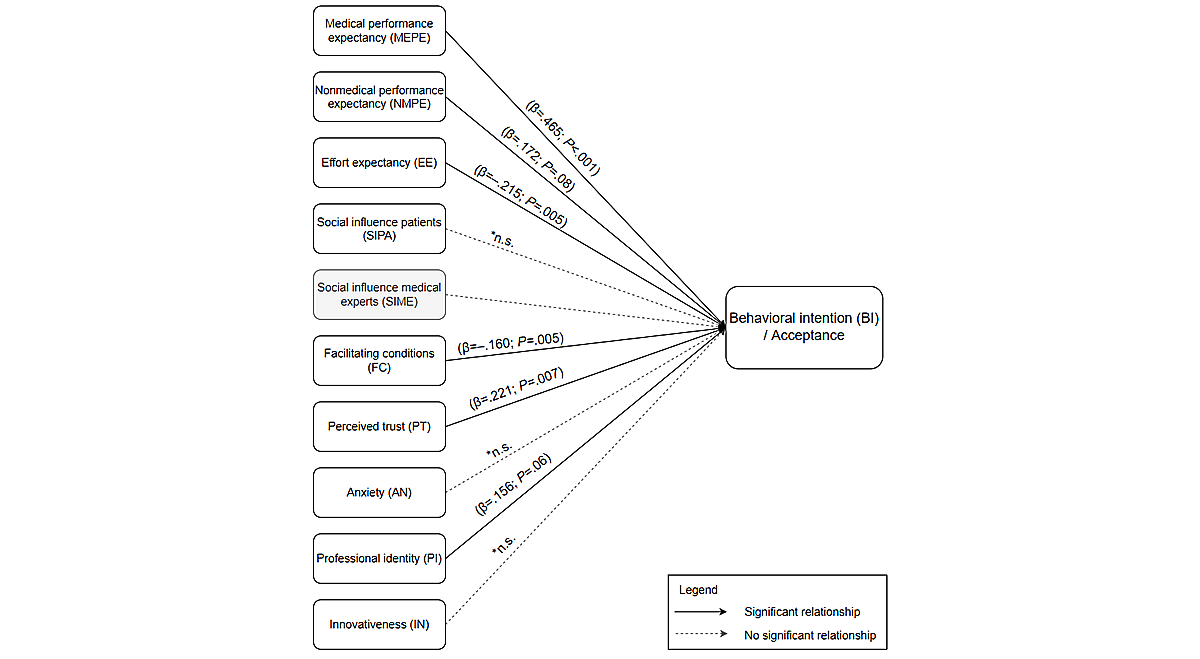
Overview of the individual relationships of the predictor variables and the acceptance of artificial intelligence–powered care pathways. Medical performance expectancy (MEPE), nonmedical performance expectancy, effort expectancy, facilitating condition, perceived trust, and professional identity showed a significantly influential relationship on acceptance where MEPE had the largest impact. Social influence patient, anxiety, and innovativeness did not show a significant relationship on the variance in acceptance. The predicator variable social influence medical was excluded from the analysis since it showed a poor internal consistency. n.s.: not significant.

The results of multiple linear regression analysis showed significant relationships between the predictor variables and the acceptance of AI-powered care pathways. Overall, the results show that 75.4% of the variance in the acceptance can be explained by the independent variables of the model (adjusted *R*^2^=0.754; *F*_9,0_=22.548; *P*<.001). From the data, it can be concluded that the model is highly significant (*P*<.001). The analysis indicated that the variables medical performance expectancy (MEPE; *β*=.465; *P*<.001), nonmedical performance expectancy (NMPE; *β*=.172; *P=*.08), PT (*β*=.221; *P=*.007), and PI (*β*=.156; *P=*.06) had a significant positive effect on the acceptance of AI-powered care pathways (Figure 2 and Multimedia Appendix 4). Both EE (*β*=–.215; *P=*.005) and FC (*β*=–.160; *P=*.005) were found to have a negative impact on acceptance. From the magnitude of the *β* statistics, MEPE had the biggest impact on variance followed by PT, EE, NMPE, FC, and PI. Some variables did not show a significant result, including SIPA (*β*=.042; *P=*.63), AN (*β*=.021; *P=*.84), and IN (*β*=.078; *P=*.30).

#### Moderating Outcomes

**Figure 3:**
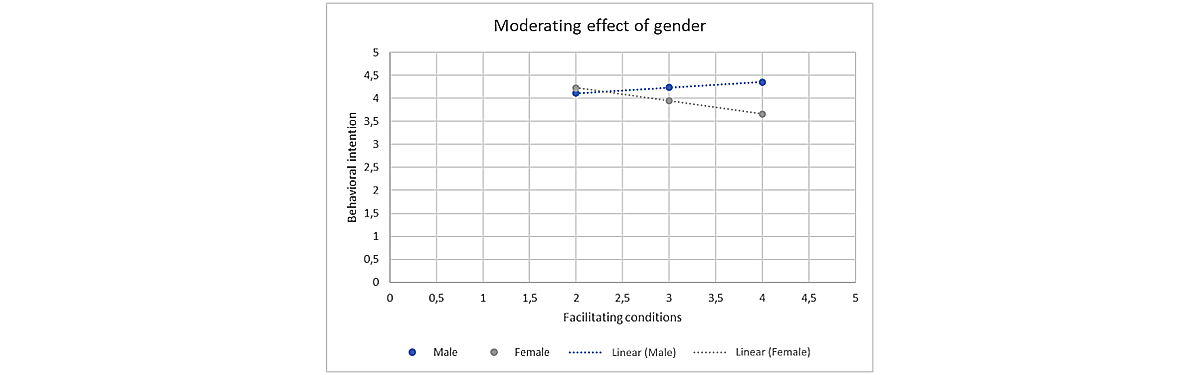
Moderating effect of gender. Being a male had a positive moderating effect whereas being a female had a negative impact.

The moderating effects of gender, age, experience, and profession were each tested for the relationships between the individual predictor variables and the acceptance to use AI-powered care pathways (Multimedia Appendix 5). Gender had a significant moderating effect on the relationship between facilitating conditions and the acceptance to use AI-powered care pathways (*β*=–.406; *P=*.09), indicating that being male had a positive moderating effect and female had a negative moderating effect (Figure 3). No other significant moderators were identified.

## Discussion

### Principal Findings

This study investigated the technology acceptance of AI-powered care pathways among medical professionals. The model explained 75.4% of the variance in acceptance of the medical professionals. The predictor variables MEPE, NMPE, EE, FC, PT, and PI were found to significantly influence the acceptance of AI-powered care pathways. SIPA, SIME, IN, and AN were not found to significantly influence the behavioral intention. One moderating relationship was found; gender moderates the relationship between FC and acceptance, with identifying as male increasing the likelihood of accepting AI-powered care pathways.

### Comparison With Prior Work

The predictor MEPE was found to have the highest impact on the acceptance of AI-powered care pathways. Several studies on the acceptance of health technology also identified performance expectancy as the main predictor [12,14,26-29]. The Predictor PEME was also found to be most important goal of physicians in the qualitative study of Lai et al [12]—they concluded that providing the best care for the patients was the main goal of physicians, and if AI-based technologies could enhance that, they were not opposed to change and the use of AI-based technologies. These findings confirm that medical professionals are more willing to use AI-powered care pathways when they see the benefits and added value considering the quality of care. Interestingly, when comparing the magnitude of the *β* values, MEPE was found to have more than double the influential strength compared to NMPE, implying that the perceived added value in terms of work efficiency, productivity, and communication has a lower impact on the acceptance than the perceived added value for the quality of care. This finding is in line with that of Shaw et al [15], where they stated that it is important to look at the value proposition between different added values a technology can bring. This finding suggests that the medical professionals in this study focus on the clinical relevance of AI-powered care pathways and that they would be more interested in the integrated care approach they would facilitate. Another possible explanation for this considerable difference in magnitude could be that medical professionals are less aware about the added value that AI-powered care pathways have regarding efficiency, productivity, and communication as most information about AI in health care tends to focus on the impact it can have on clinical aspects. This might have resulted in NMPE being less influential than MEPE in the variance of the acceptance.

A positive impact of PI was found, implying that if medical professionals perceive AI-powered care pathways as a positive stimulus to their career growth, professional status, and financial situation, they would be more willing to use the technology, and vice versa. This finding confirms the results of Jussupow et al [20], who proposed that for the successful implementations of information technology, especially AI in health care, it is crucial to identify and address professional identity threats. Currently, none of the popular technology acceptance models (UTAUT, Technology Acceptance Model, Diffusion of Innovation, and Technology Readiness Phases) include a determinant considering the influence of PI. This study highlights the importance of involving PI when studying the acceptance of target groups with a strong PI or social status and when dealing with AI-based technologies.

Social influence was not found to be an influential factor (both SIME and SIPA), which is contrary to several other studies that highlighted its importance for the adoption of new technologies [14,30]. The lack of impact of social influence in this study could be explained by the premature-stage AI-powered care pathways that the technology is in, as key opinion leaders are still absent and insufficient successful examples are present [31]. Future research should confirm if this is indeed the case. Furthermore, COVID-19 could have lowered the prioritization of AI-based technologies since the pandemic pressured the medical professionals, and no additional time was available to focus on AI-based technologies. This may have shaped their priorities and interests when they filled in the survey in ways that possibly rendered insignificant the effect of social influence. One could also argue that social influence not only shapes and helps direct the activities and approaches of medical professionals, but also is itself influenced by socioeconomic circumstances or changes along with them. During the pandemic, social influence may therefore have focused on aspects or technologies more readily directed at managing the pandemic.

This study indicated that a higher perception of the availability of FC had a negative impact on the acceptance of AI-powered care pathways, which is contrary to previous studies [32]. This implies that if medical professionals perceive the FC in their medical organization—including training and technological resources—as better, they would be less likely to accept AI-powered care pathways. This could be explained by medical professionals perceiving good FCs as a workload increase due to additional trainings and technical tasks. Interestingly, it was found that gender moderates this relationship where identifying as female had a negative moderating effect, and identifying as male a positive moderating effect. This finding is in line with that reported by Haluza and Wernhart [33], who stated that there are gender divergences that are important to incorporate when formulating a new strategy for eHealth and telemedicine implementation.

However, narrowing in on the characteristics of the specific respondent groups reveals a nonrandom sample in terms of different medical professions (Multimedia Appendix 6); all nurses included in this study identified as female, whereas physicians largely identified as male. This nonrandom sample could have influenced the moderating effect of gender given the way in which tasks and responsibilities are distributed among these professional categories. Since nurses continue to perform more administrative work compared to physicians, they may view good FC as a constraint in that it may add to their already broad repertoire of tasks, whereas physicians may view FC as a helping tool particularly given their focus on MEPE [34]. In addition, the speed of technological advances in the work field requires continuous development of new skills, which might be more challenging to cope with for nurses owing to the highly varied nature of their tasks. Therefore, they may perceive the better FC more as a demand to keep up with the fast digitalization [35,36]. Besides the difference in job-specific tasks, the differences in professional identity and distribution of professional rewards based on the acquisition of new skills could also have an influence. Traditionally, a greater focus has been placed on the need for physicians to keep up to date with the latest clinical insights and approaches, and differences in professional status and social standing have often been derived on the basis of their frequent participation to such activities. In contrast, even though important transformations have taken place over the last decades in this sense, the main task of nurses is still seen by many as the provision of care, often understood as a quality that nurses somehow naturally possess rather than a set of skills that could be trained and fostered [37]. Thus, to the extent that such trainings may not lead to obvious professional rewards, nurses may see them more as a constraint and an imposition rather than as an opportunity. Furthermore, this result may have also been influenced by the broader and often gendered realities of nurses’ lives, where family duties and other caring obligations outside their professional roles may prevent them from wanting or being able to take on new work roles and responsibilities. However, regional differences in the professional identity of nurses and physicians were found [38]. The limited sample in this study did not allow us to unambiguously prove if FC is indeed influenced by the profession or if it is mainly caused by gender. However, it is strongly suggested that both profession and gender play a role in how the perceived FC influences acceptance, so future studies should explore the relationship between these two variables and the underlying reasoning.

### Strengths and Limitations

To our knowledge, this is the first study assessing the predictors of acceptance of AI-based technologies among medical professionals, thereby contributing to a poorly understood but increasingly relevant research area. A strength of this research was that it succeeded to identify significant relationships that influence acceptance. Another strength was the successful utilization of the UTAUT model and extensions of the model. This creates a foundation for future research in the acceptance of AI-based technologies. Furthermore, the quantitative nature of this study allows for more generalizable results and facilitates comparisons with future studies.

Some limitations were present in this study. Selection bias was unavoidable since the respondents participated voluntarily on the internet, which might have resulted in more individuals with an enthusiasm and interest about AI in health care. The selection bias could have been increased by the recruitment via the physicians’ network. This might have resulted in more positive results since this network contains a lot of medical professionals with an interest in health technology.

The results revealed approximately 40% responses with missing values. Most health care professionals stopped the survey at the information page about AI-powered care pathways. This page required some reading and thus some effort to learn about AI-powered care pathways. Even though efforts made for the information provided about the AI-powered care pathways were succinct, the health care professionals may not have had the time to read these materials owing to the increased work pressure they experienced during the COVID-19 pandemic. Future iterations of this study should also interrogate respondents about the modalities through which they would be most successfully informed about these technologies when they are implemented. Visual or video materials might be more helpful when engaging with very busy professionals.

Furthermore, AI-powered care pathways are in the beginning of the implementation phase and therefore did not include the actual use behavior of AI-powered care pathways. This study could therefore not show if the acceptance is valid for predicting the actual use behavior.

Last, the used measures should be tested regarding their psychometric properties. Even though the constructs used in this study were mainly based upon validated models, the usefulness in the context of AI-powered care pathways needs to be further investigated. In addition, new constructs were added and some constructs were adjusted, which requires further investigation. The internal consistency of the constructs was tested with the Cronbach *α*. Two constructs (FC and SIME) showed low internal consistency. FC was still included owing to the exploratory nature; we did consider lower Cronbach *α* values (<.5) since it adds critical information to the research. The internal consistency of SIME did not allow for its inclusion in deregression. This is a limitation since this study misses a potential predictor and it could still have contributed to nonzero amounts to the explained variance in the case of correlated regressors, which can be done by influencing other significant regressors.

### Future Implications

This research should function as a foundation for future longitudinal research. Future research could identify if acceptance differs over adoption steps and when more awareness about the technology is present. This study was conducted in quite a premature stage where actual use is still limited.

Furthermore, future research should identify if the used model is applicable in different health care systems or in other regions of the world. Since this research was conducted in the Netherlands and included all type of medical organizations, variations between organizational cultures, differences in professional identity, and the difference in public opinion about AI were not taken into account. Insight into these differences could help develop adequate implementation strategies per region and organization.

Adaptations were made to make the model fit better to the research aim. Future studies should focus on further validating the model in the context of AI-based technologies, especially the construct with poor internal consistency.

Since performance expectancy was found as the strongest predictor for the acceptance of AI-powered care pathways, this should be high priority during implementation of AI-based health technologies. The added value of these technologies should be clearly communicated to the end users. PT was the second most influencing variable for the acceptance of AI-powered care pathways. Strategies on how to increase trust in AI-based technologies should therefore be formulated for successful adoption in health care. Even though trust is found to be an important facilitator for acceptance, future research should not only focus on how to increase trust but also what effect this trust has on the actual use, since studies found that people tend to overtrust and misinterpret the outcomes of AI-based decision support [39-41].

The quantitative nature of this study did not allow us to understand the medical professionals’ reasoning underlying the found outcomes. Future qualitative studies are therefore recommended to understand how specific personality traits, the amount of understanding of AI-powered care pathways, or other contextual factors influence the acceptance of AI-based technologies.

### Conclusions

This study sheds light on what factors have the largest impact on the acceptance of AI-powered care pathways among hospital staff and medical professionals. The model explained 75.4% of the variance in the behavioral intention. MEPE, NMPE, EE, PT, and PI were found to significantly influence behavioral intention where medical performance expectancy was found to have the largest impact. The moderator gender was found to significantly influence the relationship between facilitating conditions and acceptance. Since this study was conducted among Dutch medical professionals over a limited period of time and at a stage where the implementation of these technologies is still limited, follow-up surveys and multinational studies could further explore the predictors of acceptance of AI-powered care pathways over time and in different context.

## Appendix

Multimedia Appendix 1 Survey items with the corresponding item sources.

Multimedia Appendix 2 Graphs and tables for assumption testing multiple linear regression.

Multimedia Appendix 3 Pearson correlations between the variables.

Multimedia Appendix 4 Results from the multiple linear regression indicating the relationship between the predictor variables and the behavioral intention to use AI-powered care pathways.

Multimedia Appendix 5 Hayes’ PROCESS regression matrix for the moderating effects on the relationships between the predictor variables and the behavioral intention to use AI-powered care pathways. The Coefficient, standard error and P-value of the interaction terms are shown.

Multimedia Appendix 6 Cross Table for gender x profession.

## Abbreviations

AI: artificial intelligence
AN: anxiety
BI: behavioral intention
EE: effort expectancy
FC: facilitating condition
IN: innovativeness
MEPE: medical performance expectancy
NMPE: nonmedical performance expectancy
PI: professional identity
PT: perceived trust
SI: social influence
SIME: social influence medical experts
SIPA: social influence patients
UTAUT: Unified Theory of Acceptance and Use of Technology
