# Supplementary material for: The Drivers of Acceptance of Artificial Intelligence–Powered Care Pathways Among Medical Professionals: Web-Based Survey Study
Source: JMIR Form Res. 2022 Jun 21;6(6):e33368. doi: 10.2196/33368 (PMC9384807; doi:10.2196/33368)
Supplement: Multimedia Appendix 1 [file formative_v6i6e33368_app1.doc]

**Multimedia Appendix 1**: Survey items with the corresponding item sources

| **Construct** | | **Item** | **Item source** |
| --- | --- | --- | --- |
| **MEPE** |  | |  |
| MEPE1 | I think the use of AI-powered care pathways would improve the overview of disease control over the patient population. | | New item |
| MEPE2 | I think the use of AI-powered care pathways enables better communication between patient and healthcare professional. | | New item |
| MEPE3 | I think the recommendations given by AI-powered care pathways can support a beneficial and diverse set of approaches to treatments. | | New item |
| **NMPE** |  | |  |
| NMPE1 | I think using AI-powered care pathways would enable me to accomplish my tasks more efficiently. | | New item |
| NMPE2 | I think the use of AI-powered care pathway results in less administrative work for me. | | New item |
| NMPE3 | I think the use of AI-powered care pathways results in better communication between medical professionals. | | New item |
| **EE** |  | |  |
| EE1 | I believe I have the skills to use AI-powered care pathways. | | [13] |
| EE2 | I think working with AI-powered care pathways is easy to understand. | | [13] |
| EE3 | I think it would not take me long to learn how to work with AI-powered care pathways. | | [13] |
| **SIME** |  | |  |
| SIME1 | I think other organizations/departments that already work with AI-powered care pathways have improved their way of working. | | New item |
| SIME2 | I know colleagues who already have positive experiences with AI-powered care pathways. | | Adapted from [13] |
| SIME3 | Colleagues have advised me to start working with AI-powered care pathways. | | Adapted from [13] |
| **SIPA** |  | |  |
| SIPA1 | I think that in general my patients will be enthusiastic to use AI-powered care pathways. | | New item |
| SIPA2 | I think most of my patients want to be included in the use of AI-powered care pathways. | | New item |
| SIPA3 | I think my patients will positively perceive me working with AI-powered care pathways. | | Adapted from [13] |
| **FC** |  | |  |
| FC1 | I think the necessary technical preconditions are present in my medical organization for the use of AI-powered care pathways. | | [13] |
| FC2 | I believe the medical institution where I work will provide the necessary training and information so that I can successfully use AI-powered care pathways. | | [13] |
| FC3 | I think AI-powered care pathways are compatible with the other technologies that I use. | | (13) |
| **PT** |  | |  |
| PT1 | I trust that all sensitive data in the AI-powered care pathways will be stored and processed confidentially. | | New item |
| PT2 | I trust that recommendations from the AI-powered care pathway are reliable. | | New item |
| PT3 | I trust that the AI-powered care pathways are programmed to respect the integrity of the patient. | | New item |
| **AN** |  | |  |
| AN1 | I feel comfortable about the use of artificial intelligence in my daily practice. | | New item |
| AN2 | I feel confident that I understand/ how the recommendations provide by AI-powered care pathways are produced. | | New item |
| AN3 | I feel confident that AI-powered care pathways will not intervene with my ethical obligations/ moral obligations against patients. | | New item |
| **PI** |  | |  |
| PI1 | I think the use of AI-powered care pathways will negatively affect my salary/income. | | New item |
| PI2 | I think that the use of AI-powered care pathways will negatively affect my professional status. | | New item |
| PI3 | I think the use of AI-powered care pathways will negatively affect my career growth. | | New item |
| **IN** |  | |  |
| IN1 | Among my peers, I am usually the first to try out new technologies. | | New item |
| IN2 | I like to experiment with new technologies. | | [17] |
| IN3 | I am competent to make new technologies work in the way I expect them to. | | [17] |
| **BI** |  | |  |
| BI1 | I would like to use AI-powered care pathways. | | [13] |
| BI2 | I would be willing to use AI-powered care pathways. | | [13] |
| BI3 | I think we should use AI-powered care pathways. | | [13] |
