# Supplementary material for: The Drivers of Acceptance of Artificial Intelligence–Powered Care Pathways Among Medical Professionals: Web-Based Survey Study
Source: JMIR Form Res. 2022 Jun 21;6(6):e33368. doi: 10.2196/33368 (PMC9384807; doi:10.2196/33368)
Supplement: Multimedia Appendix 2 [file formative_v6i6e33368_app2.docx]

**Multimedia Appendix 2**


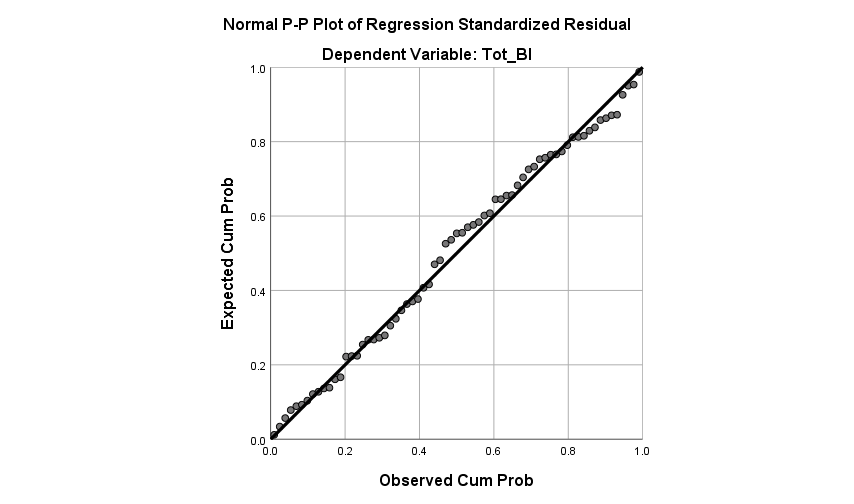


Figure 1: P-P plot showed a linear relationships


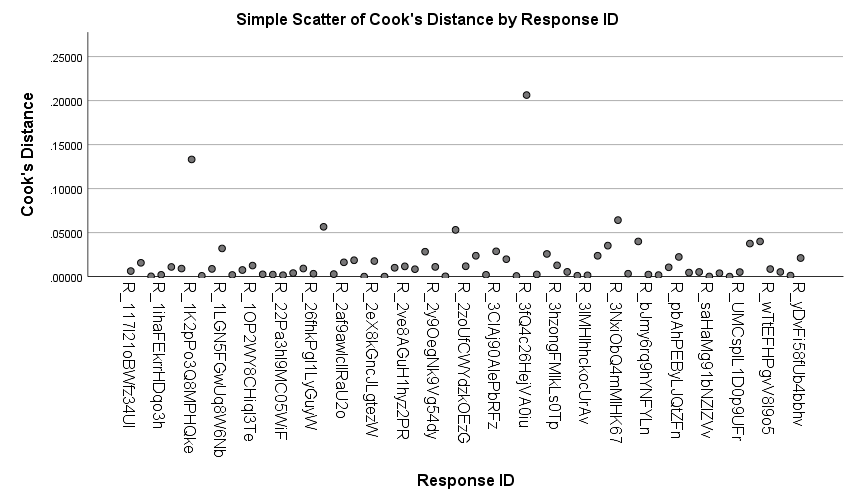


Figure 2:Cook’s distances with the different response ID’s


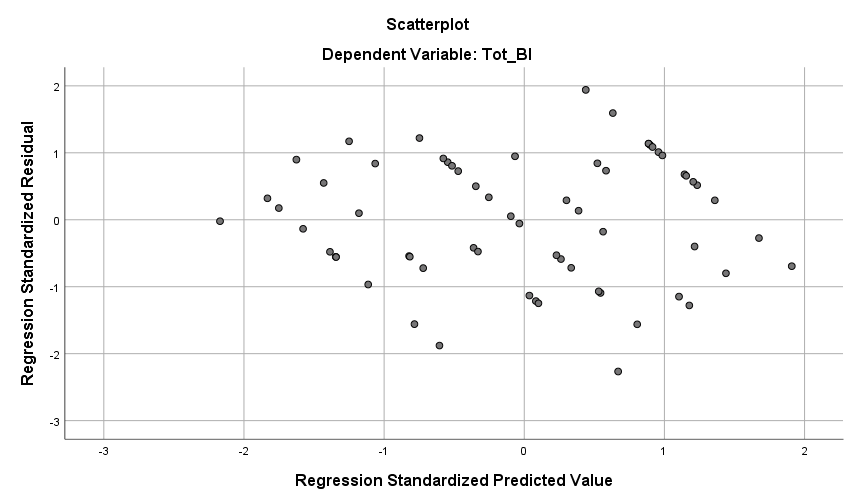


Figure 3: Scatterplot to check for heteroscedasticity.


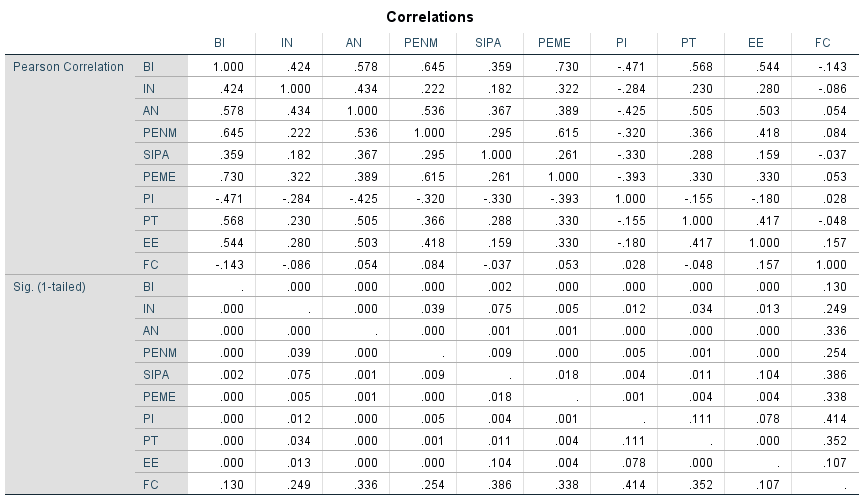


Figure 4: Pearson correlations between predictors.


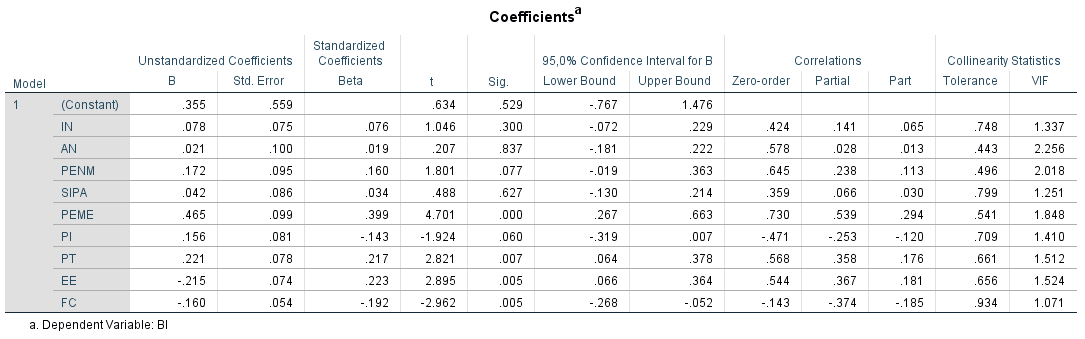


Figure 5: In this table the collinearity statistics (Tolerance and VIF) are shown for the predictor variables.


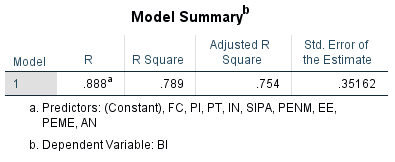


Figure 6: Model summary of the multiple linear regression


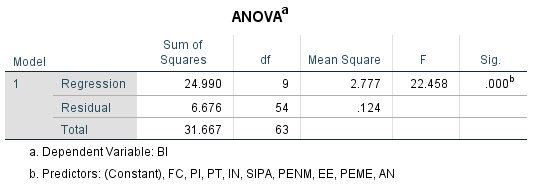


Figure 7: ANOVA table that show the significance of the model.
