# Supplementary material for: The Drivers of Acceptance of Artificial Intelligence–Powered Care Pathways Among Medical Professionals: Web-Based Survey Study
Source: JMIR Form Res. 2022 Jun 21;6(6):e33368. doi: 10.2196/33368 (PMC9384807; doi:10.2196/33368)
Supplement: Multimedia Appendix 3 [file formative_v6i6e33368_app3.doc]

**Multimedia Appendix 3**

**Table:** Pearson correlations between the variables.

|  | BI | IN | AN | NMPE | SIPA | MEPE | PI | PT | EE | FC |
| --- | --- | --- | --- | --- | --- | --- | --- | --- | --- | --- |
|  |  |  |  |  |  |  |  |  |  |  |
| BI | 1.000 |  |  |  |  |  |  |  |  |  |
| IN | .424 | 1.000 |  |  |  |  |  |  |  |  |
| AN | .578 | .434 | 1.000 |  |  |  |  |  |  |  |
| NMPE | .645 | .222 | .536 | 1.000 |  |  |  |  |  |  |
| SIPA | .359 | .182 | .367 | .295 | 1.000 |  |  |  |  |  |
| MEPE | .730 | .322 | .389 | .615 | .216 | 1.000 |  |  |  |  |
| PI | -.471 | -.284 | -.425 | -.320 | -.330 | -.393 | 1.000 |  |  |  |
| PT | .568 | .230 | .505 | .366 | .288 | .330 | .-155 | 1.000 |  |  |
| EE | .544 | .280 | .503 | .418 | .159 | .330 | -.180 | .417 | 1.000 |  |
| FC | -.143 | -.086 | .054 | 0.048 | -.037 | .053 | .028 | -.048 | .157 | 1.000 |
