# Supplementary material for: The Drivers of Acceptance of Artificial Intelligence–Powered Care Pathways Among Medical Professionals: Web-Based Survey Study
Source: JMIR Form Res. 2022 Jun 21;6(6):e33368. doi: 10.2196/33368 (PMC9384807; doi:10.2196/33368)
Supplement: Multimedia Appendix 4 [file formative_v6i6e33368_app4.doc]

**Multimedia appendix 4**

Results from the multiple linear regression indicating the relationship between the predictor variables and the behavioral intention to use AI-powered care pathways.

| Predictor variables | beta | SE | t-value | Sig. |
| --- | --- | --- | --- | --- |
|  |  |  |  |  |
| MEPE🡪 BI | 0.465 | 0.099 | 4.701 | .000** |
| NMPE🡪 BI | 0.172 | 0.095 | 1.801 | .077* |
| EE🡪BI | -0.215 | 0.074 | 2.895 | .005** |
| SIME🡪BI | - | - | - | - |
| SIPA🡪BI | 0.042 | 0.086 | 0.488 | .627 |
| FC🡪BI | -0.160 | 0.054 | -2.962 | .005** |
| PT🡪BI | 0.221 | 0.078 | 2.821 | .007** |
| AN🡪BI | 0.021 | 0.100 | 0.207 | .837 |
| PI🡪BI | 0.156 | 0.081 | -1.924 | .060* |
| IN🡪BI | 0.078 | 0.075 | 1.046 | .300 |

**P<0,1 **P <0,05*
