# Supplementary material for: The Drivers of Acceptance of Artificial Intelligence–Powered Care Pathways Among Medical Professionals: Web-Based Survey Study
Source: JMIR Form Res. 2022 Jun 21;6(6):e33368. doi: 10.2196/33368 (PMC9384807; doi:10.2196/33368)
Supplement: Multimedia Appendix 5 [file formative_v6i6e33368_app5.doc]

**Multimedia Appendix 5:**

Hayes’ PROCESS regression matrix for the moderating effects on the relationships between the predictor variables and the behavioral intention to use AI-powered care pathways. The Coefficient, standard error and P-value of the interaction terms are shown.

|  |  | Age | Gender | Experience | Profession |
| --- | --- | --- | --- | --- | --- |
|  |  |  |  |  |  |
| MEPE | Coef | -.012 | .216 | -.0216 | .049 |
|  | SE | .0825 | .1988 | .0807 | .0518 |
|  | *P* | .8849 | .2817 | .7896 | .348 |
| NMPE | Coef | .0255 | .0141 | .0178 | -.0211 |
|  | SE | .0781 | .2041 | .0652 | .0571 |
|  | *P* | .7746 | .9453 | .7854 | .713 |
| EE | Coef | .0335 | .099 | .0377 | .0045 |
|  | SE | .0798 | .2072 | .0701 | .0557 |
|  | *P* | .6764 | .6343 | .5931 | .9362 |
| SIME | Coef | .1382 | .1461 | .1125 | .1125 |
|  | SE | .1014 | .2414 | .0947 | .0947 |
|  | *P* | .178 | .5475 | .2399 | .2399 |
| SIPA | Coef | .0381 | -.3753 | .043 | .1125 |
|  | SE | .1193 | .2919 | .089 | .0981 |
|  | *P* | .7503 | .206 | .6307 | .2565 |
| FC | Coef | 645 | -.796 | .1239 | .0166 |
|  | SE | .0731 | .2693 | .0748 | .0593 |
|  | *P* | .3813 | .0044 | .1026 | .781 |
| PT | Coef | -.0323 | .0968 | .0213 | -.0294 |
|  | SE | .0715 | .2154 | .0673 | .0603 |
|  | *P* | .6529 | .6547 | .7533 | .6276 |
| AN | Coef | -.0088 | .093 | -.0359 | .0214 |
|  | SE | .0873 | .2388 | .0808 | .0753 |
|  | *P* | .9202 | .6982 | .6582 | .7767 |
| PI | Coef | -.0086 | -.0137 | .0004 | .0056 |
|  | SE | .0847 | .2547 | .0822 | .0807 |
|  | *P* | .9193 | .9573 | .9966 | .9451 |
| IN | Coef | -.0562 | .1902 | -.0885 | -.1069 |
|  | SE | .0989 | .2859 | .0936 | .066 |
|  | *P* | .5723 | .5085 | .3481 | .1106 |
