# Supplementary material for: The Drivers of Acceptance of Artificial Intelligence–Powered Care Pathways Among Medical Professionals: Web-Based Survey Study
Source: JMIR Form Res. 2022 Jun 21;6(6):e33368. doi: 10.2196/33368 (PMC9384807; doi:10.2196/33368)
Supplement: Multimedia Appendix 6 [file formative_v6i6e33368_app6.doc]

**Multimedia Appendix 6**

Cross Table for gender x profession

| Profession | Gender | | Total |
| --- | --- | --- | --- |
|  |  |  |  |
|  | Male | Female |  |
|  |  |  |  |
| Physician | 16 | 12 | 28 |
| Nurse | 0 | 15 | 15 |
| Management | 3 | 6 | 9 |
| Consultant | 4 | 4 | 4 |
| Other | 3 | 4 | 7 |
|  |  |  |  |
| Total | 26 | 41 | 67 |
